# Author Correction: Reovirus directly engages integrin to recruit clathrin for entry into host cells

**DOI:** 10.1038/s41467-021-23537-7

**Published:** 2021-05-13

**Authors:** Melanie Koehler, Simon J. L. Petitjean, Jinsung Yang, Pavithra Aravamudhan, Xayathed Somoulay, Cristina Lo Giudice, Mégane A. Poncin, Andra C. Dumitru, Terence S. Dermody, David Alsteens

**Affiliations:** 1 Louvain Institute of Biomolecular Science and Technology, Université catholique de Louvain, Louvain-la-Neuve, Belgium; 2 Department of Pediatrics, University of Pittsburgh School of Medicine, Pittsburgh, PA USA; 3 Institute of Infection, Inflammation and Immunity, UPMC Children’s Hospital of Pittsburgh, Pittsburgh, PA USA; 4 Department of Microbiology and Molecular Genetics, University of Pittsburgh School of Medicine, Pittsburgh, PA USA; 5 Walloon Excellence in Life sciences and Biotechnology (WELBIO), Wavre, Belgium

**Keywords:** Nanoscale biophysics, Virus-host interactions, Applications of AFM

Correction to: *Nature Communications* 10.1038/s41467-021-22380-0, published online 12 April 2021.

The original version of this Article omitted the following from the Acknowledgements: “The cartoon in Figure 7d was created with BioRender.com”. This has now been corrected in both the PDF and HTML versions of the Article.

