# Supplementary material for: Reovirus directly engages integrin to recruit clathrin for entry into host cells
Source: Nat Commun. 2021 Apr 12;12:2149. doi: 10.1038/s41467-021-22380-0 (PMC8041799; doi:10.1038/s41467-021-22380-0)
Supplement: Supplementary file 1 — Supplemenatry information [file 41467_2021_22380_MOESM1_ESM.pdf]

Supplementary Information for

Reovirus directly engages integrin to recruit clathrin for entry into host cells

Koehler *et al.*

This PDF file includes:

Supplementary Table 1

Supplementary Figures 1 to 8

Other Supplementary Materials for this manuscript includes the following:

Supplementary Movies 1 to 7

Supplementary Table 1| PCR primers for engineering mutations in L2.

Mutant	Fragment 1 primers (5'-3')	Fragment 2 primers (5'-3')
TGA/TGA	*Fwd: ACAGGCGCCATCGTCACTTGCATGCTG*<br>*Rev: GGCTCCTGTGTTAGCATCAAACTGAGCC*	*Fwd: GCTAACACAGGAGCCTGGTCATTGGACATGGTGTTC*<br>*Rev: GACGATGGCGCCTGTTATACCCGTTATCCAACCGTCAC*
RGA/KGA	*Fwd: GCCATCGTCACTTGCATGCTGTCTC*<br>*Rev: GGCTCCTTTGTTAGCATCAAACTGAGCC*	*Fwd: TGCTAACAAAGGAGCCTGGTCATTGGACATGGTGTTC*<br>*Rev: GCAAGTGACGATGGCGCCACGTATACCCGTTATCC*

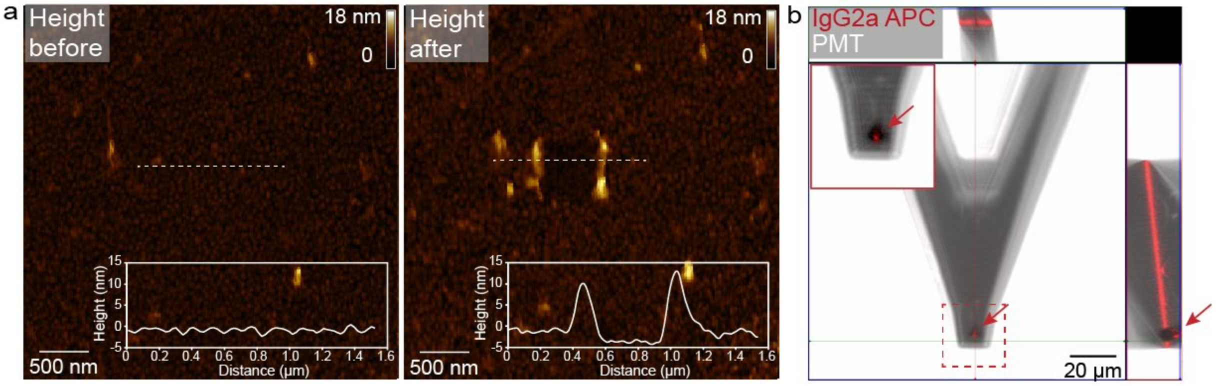

Supplementary Figure 1| Validation of surface characteristics and virus immobilization on AFM tip. (**a**) AFM topography image of an integrin-coated surface before (left) and after (right) scanning a 500 x 500 nm area in the center at a high force (~ 18 nN) to remove attached molecules (referred to as the “scratching” experiment). Insets: Plot of surface thickness along the white dashed line before and after scratching. The extracted profile after scratching shows an accumulation of material along the sides of the scratched square. The biomolecule-free surface inside the square was ~ 3 nm lower than the surrounding biomolecule-coated surface, providing an estimate of the integrin coating thickness. (**b**) Image of an AFM tip functionalized with reovirus obtained using laser-scanning optical microscopy after staining with a primary antibody against reovirus and an APC-conjugated secondary antibody (red). The inset image shows the virion at the tip apex. Experiments were repeated three times and yielded similar results.

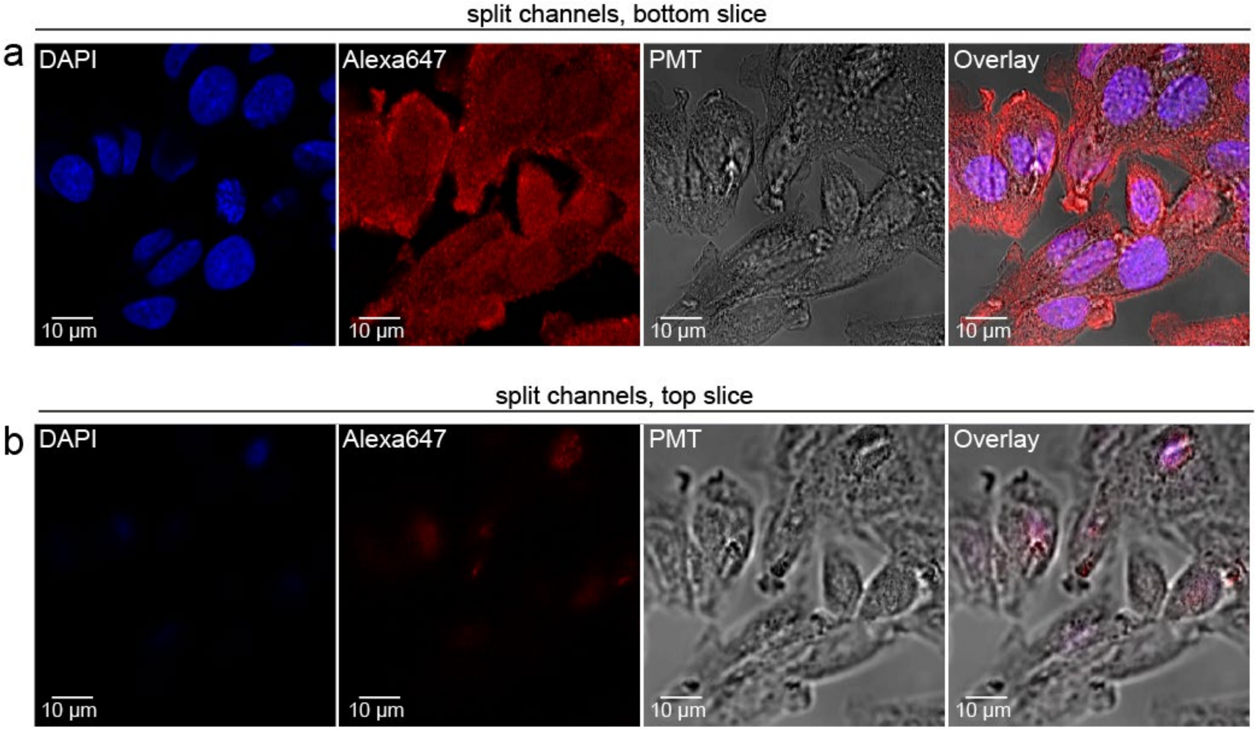

Supplementary Figure 2| Validation of integrin expression in Lec2 cells. Confocal microscopy of Lec2 cells stained for integrin expression (incubation with antibody against α5β1 integrin followed a secondary antibody conjugated with Alexa Fluor 647; red) and nuclei stained using DAPI (blue). Integrins are mainly located at the (**a**) bottom slice of the cells rather than on the (**b**) top slice. This experiment was repeated independently (N = 3) with similar results.

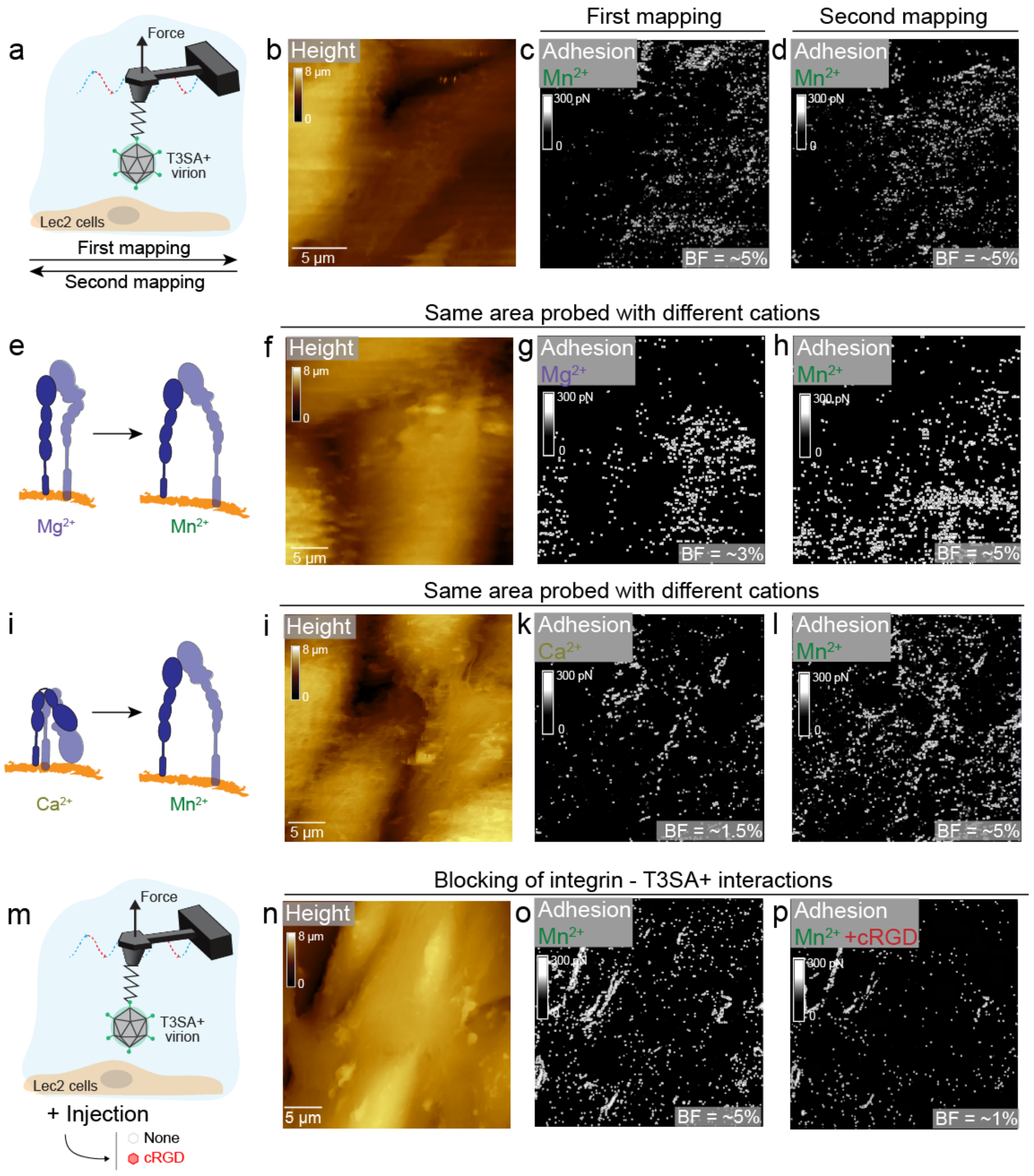

Supplementary Figure 3| Control experiments for studying the contribution of integrin in reovirus binding to living cells. (**a-d**) Consecutive probing of T3SA+ virus on Lec2 cells show similar results. (**a**) Schematic depicts the experimental setup. Height image (**b**) and adhesion probability measured in the corresponding area using FD-based AFM show similar results during two consecutive scans (**c, d**), indicating that the virus was firmly attached to the tip and did not degrade over time. (**e-l**) Consecutive probing of T3SA+ binding to cells in the presence of different

cations. (**e**) Schematic depicts the experimental setup (**e**). FD-based AFM was used to image the height (**f**) and obtain adhesion forces in the corresponding area of Lec2 cells probed first in the presence of Mg^{2+} (intermediate affinity) (**g**) and subsequently in the presence of Mn^{2+} (high affinity) (**h**). (**i**) Schematic depicts the experimental setup. FD-based AFM was used to image the height (**j**) and obtain adhesion forces in the corresponding area of Lec2 cells probed with T3SA+ virions first in the presence of Ca^{2+} (low affinity) (**k**) and subsequently in the presence of Mn^{2+} (high affinity) (**l**). A significant increase in adhesion forces (white pixels) on Lec2 cells after exchanging the divalent cations from either Mg^{2+} or Ca^{2+} to Mn^{2+} supports observations using model surfaces that β1 integrin binds reovirus in a cation-dependent manner. (**m-p**) To test the specificity of T3SA+ interaction with integrin, experiments were conducted in the presence of an integrin blocking peptide (cRGD). (**m**) Schematic depicts the experimental setup. (**n**) FD-based AFM was used to image the height and obtain adhesion forces in the corresponding area of Lec2 cells probed with T3SA+ virions first in the absence of cRGD (**n, o**), and subsequently after injection of 2 mM cRGD (**p**). A significant reduction in adhesion events can be observed following addition of cRGD. All AFM images were acquired using an oscillation frequency of 0.25 kHz and amplitude of 750 nm, under cell culture conditions. Experiments were repeated 5 to 10 times. For higher visibility, the pixel size in the adhesion image was enlarged two-fold.

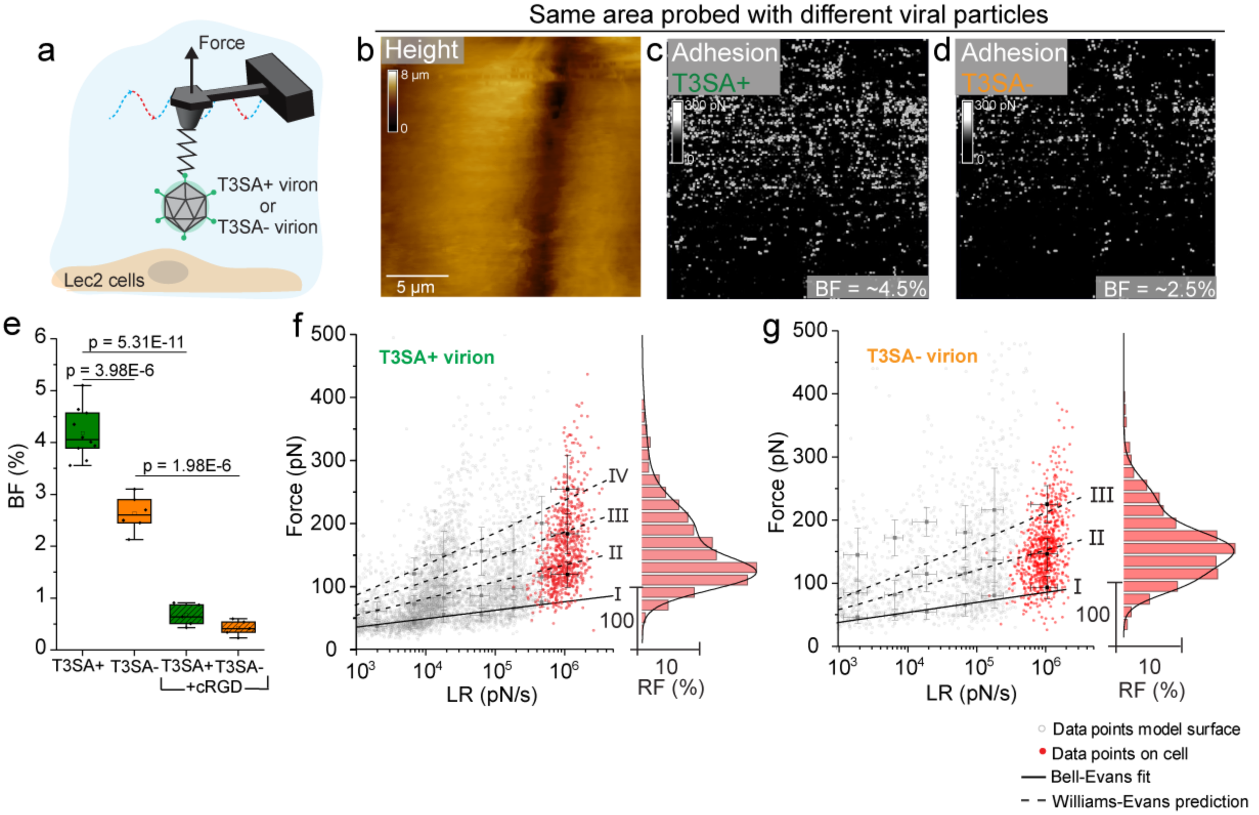

Supplementary Figure 4| Alterations to the sialic acid binding site of reovirus σ1 attachment protein changes virus interaction with β1 integrin on living cells. (**a-d**) The same area on a cell was probed sequentially with T3SA+ and then T3SA- virions. (**a**) Schematic depicts the experimental setup. (**b**) FD-based AFM was used to image the height and obtain adhesion forces in the corresponding area probed first with T3SA+ virions on the tip (**c**), followed by T3SA- virions on the tip (**d**). A significant decrease in adhesion event frequency (white pixels) on Lec2 cells after changing the tip to the non-SA-binding virus supports results observed using the model surface (Fig. 4 a-e). (**e**) Box plot of BF of T3SA+ (green) or T3SA- (yellow) virions to β1 integrins expressed on living Lec2 cells in the presence of Mn^{2+} and following injection of cRGD (dashed boxes). The horizontal line within the box indicates the median, boundaries of the box indicate the 25th and 75th percentile, and whiskers indicate the highest and lowest values of the results. An open square within each box indicates the mean. (**f, g**) Reovirus establishes multiple bonds with integrin expressed on living cells in a sialic acid-dependent manner. DFS plots show data obtained using β1 integrin-coated model surfaces (grey circles, from Fig. 3b or Fig. 4c) or living cells (red dots) probed with either T3SA+ virions (**f**) or T3SA- virions (**g**). Histogram of the force distribution observed on cells and a multi-peak Gaussian fit of data (N = 900 data points) are shown on the right side. Error bars indicate s.d. of the mean value. All AFM images were acquired using an oscillation frequency of 0.25 kHz and amplitude of 750 nm, under cell culture conditions. All data are representative of N=5 independent experiments. P values were determined by two-sample *t*-test using Origin.

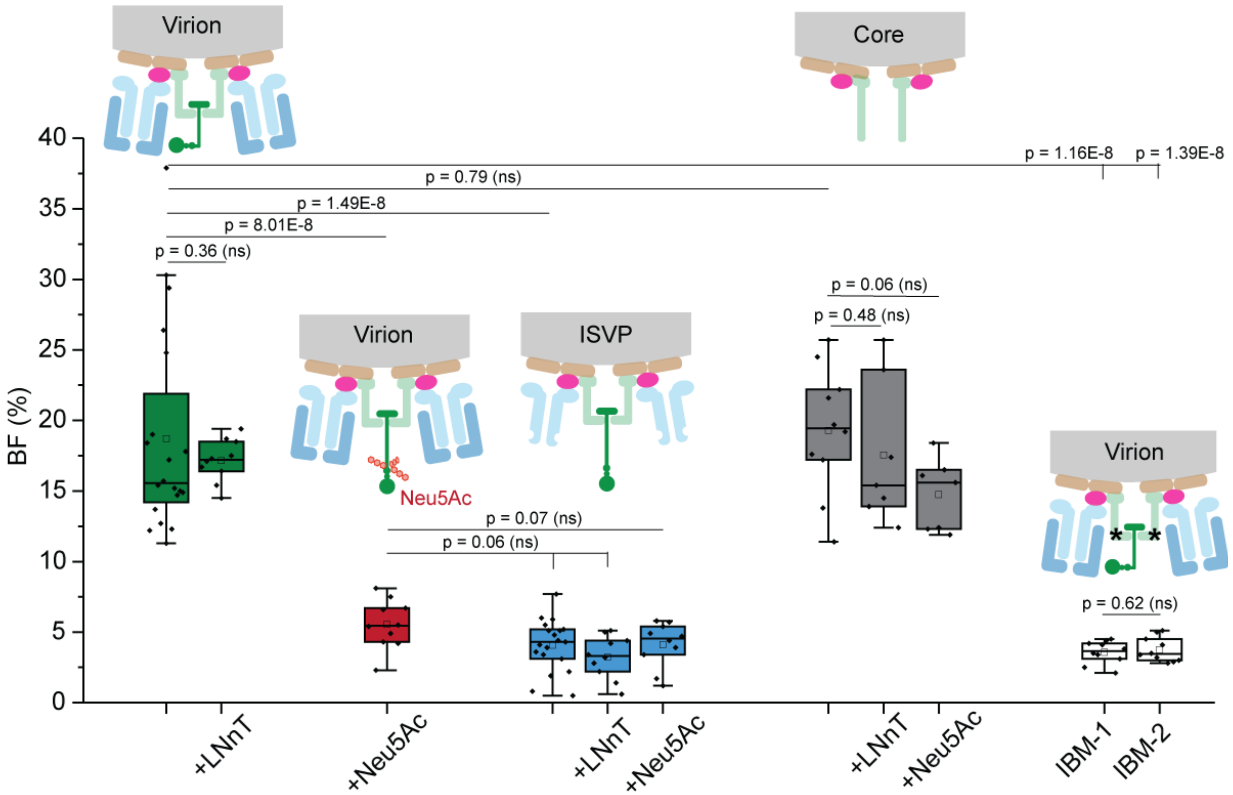

Supplementary Figure 5| Binding probabilities of T3SA+ reovirus disassembly intermediates and mutants to β1 integrin are consistent with λ2 being the integrin ligand. Box plot shows BF of integrin to T3SA+ virions (green or red), ISVPs (blue), or cores (grey) in the absence or presence of 1 mM LnNT (glycan lacking SA group) or 1 mM Neu5Ac (glycan with SA group). BF of β1 integrin to T3SA+ integrin binding motif (IBM) mutants is shown in white boxes. All experiments were conducted in the presence of the highest β1 integrin activator, Mn^{2+}. The horizontal line within each box indicates the median, boundaries of the box indicate the 25th and 75th percentiles, and whiskers indicate the highest and lowest values. An open square within each box indicates the mean. Error bars indicate s.d. of the mean value. All data are representative of N=3 independent experiments and 10 tips in total were tested per condition. P values were determined by two-sample t-test using Origin (ns = not significant, P > 0.05).

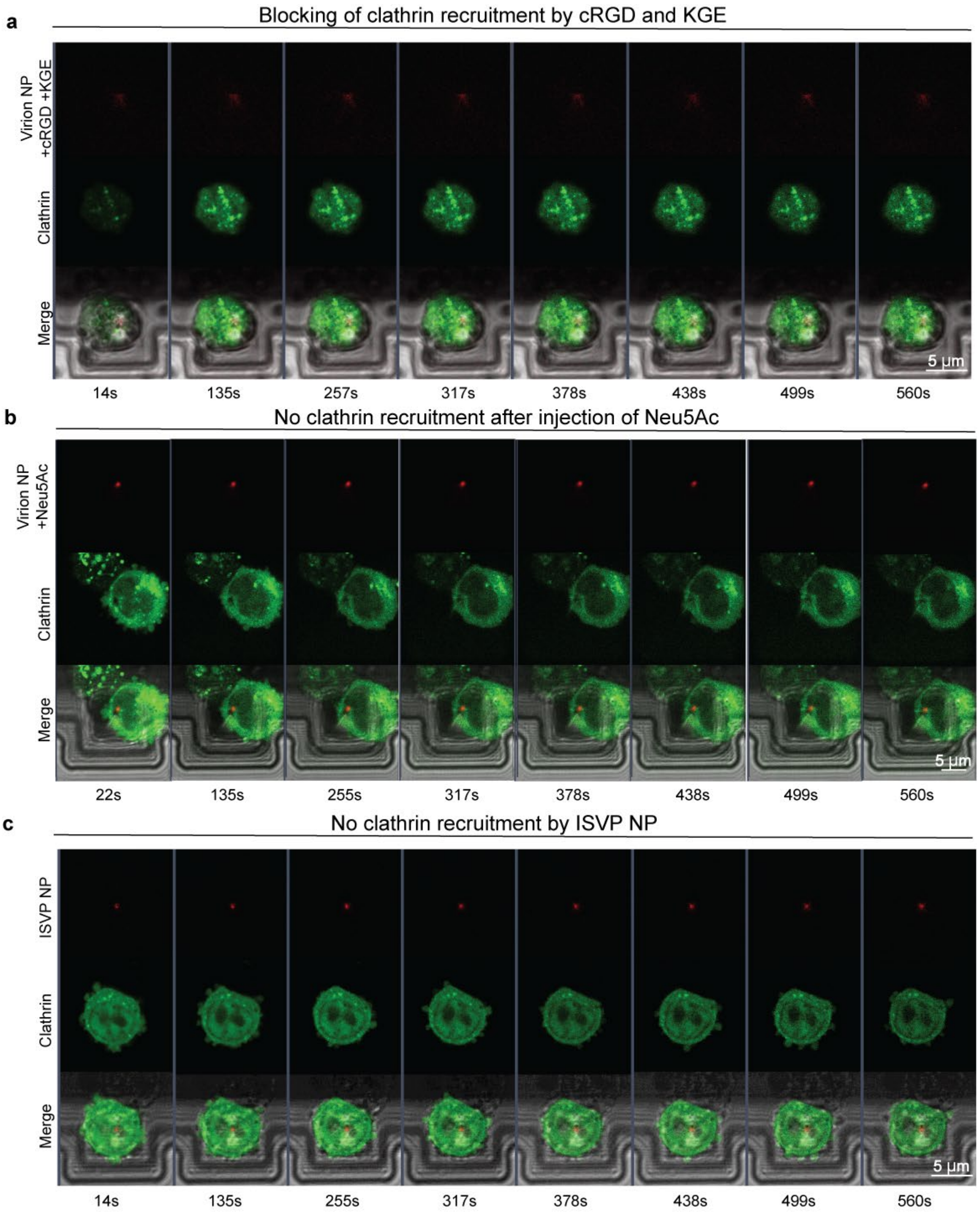

Supplementary Figure 6| Disruption of reovirus-integrin interaction abrogates clathrin recruitment to the plasma membrane. Representative time-lapse images were obtained using Fluid-FM coupled with confocal microscopy. Cells were probed with NPs decorated with T3SA+ virions either in the presence of integrin blocking peptides KGE and cRGD (**a**) or after injecting Neu5Ac, which induces an extended conformation of $\sigma1$ (**b**). The latter is similar to the $\sigma1$ conformation in the ISVP particle, which also does not recruit clathrin (**c**). These experiments were repeated independently (N = 3) with similar results.

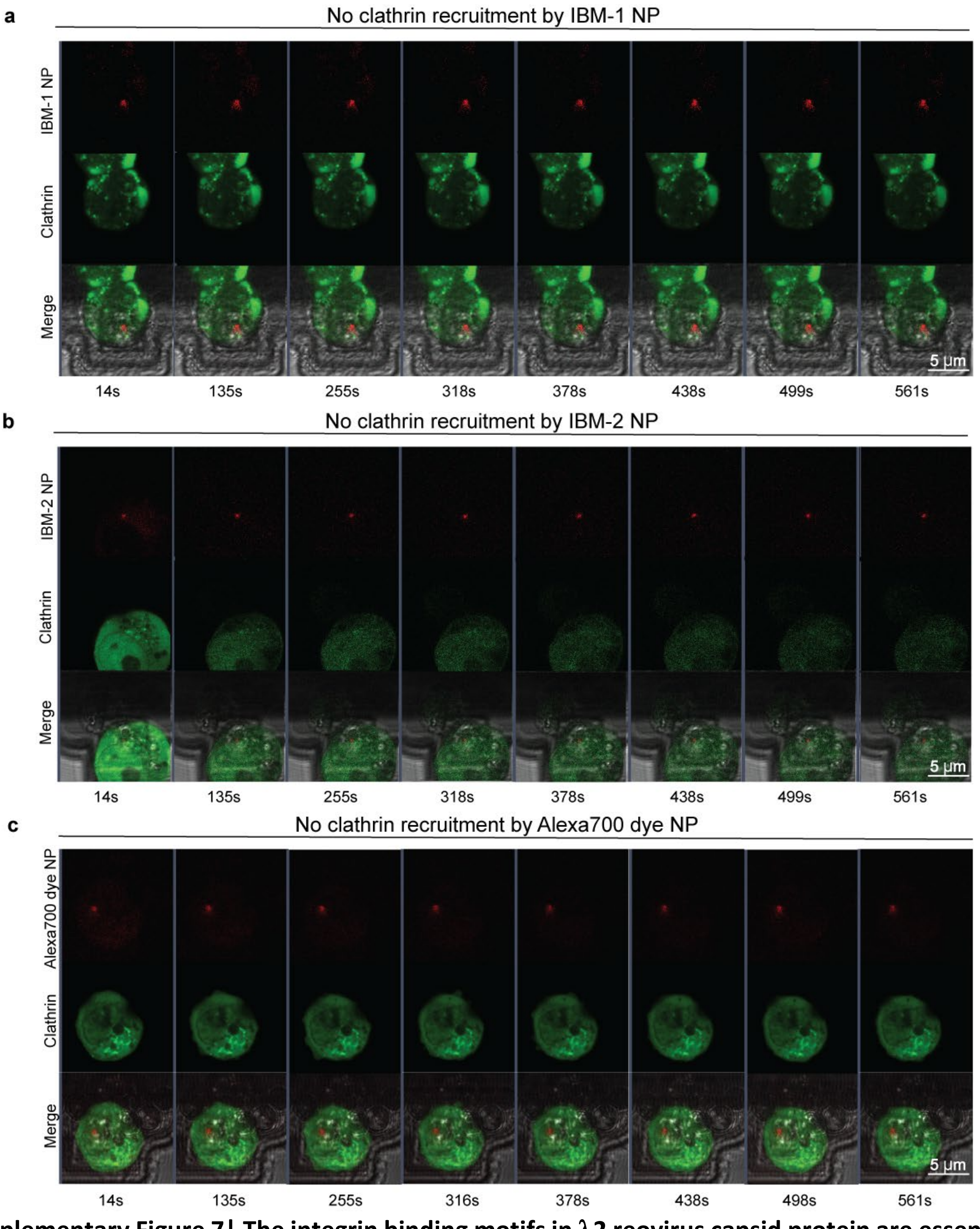

Supplementary Figure 7| The integrin binding motifs in λ2 reovirus capsid protein are essential to recruit clathrin to plasma membrane. Representative time-lapse images were obtained using Fluid-FM coupled with confocal microscopy. Cells were probed with NPs decorated with (**a**) T3SA+ IBM-1, (**b**) T3SA+ IBM-2 or (**c**) Alexa700 dye only. These experiments were repeated independently (N = 3) with similar results.

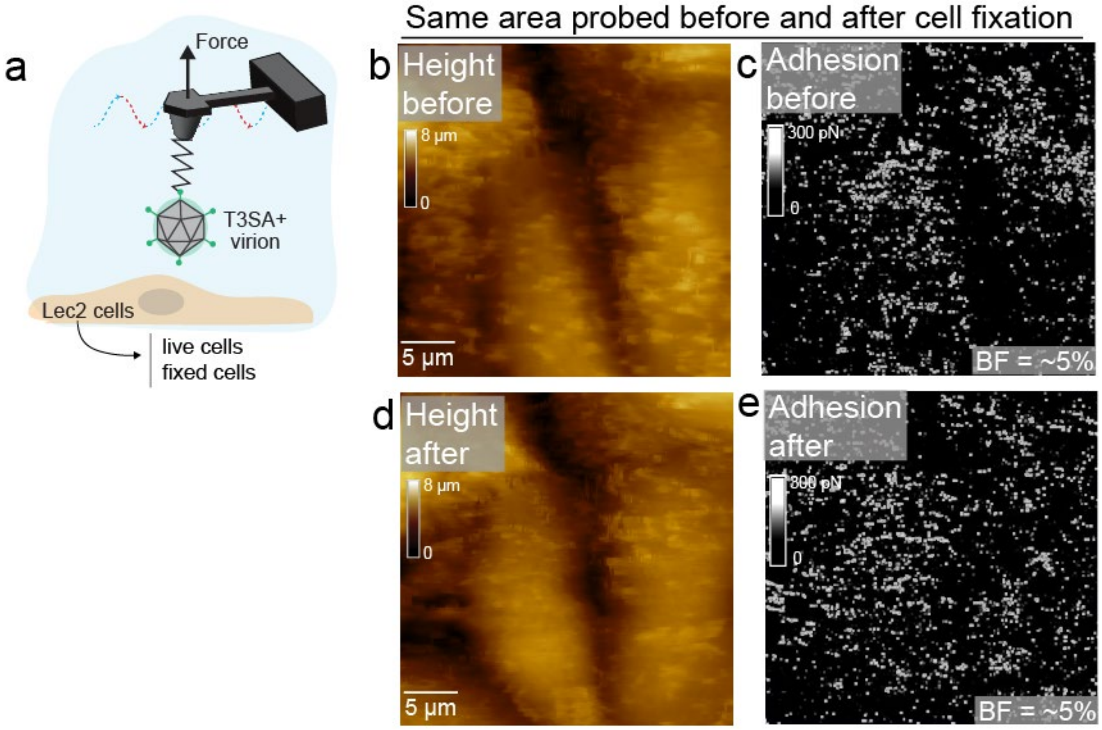

Supplementary Figure 8|Comparison of virus binding to cell surface before and after fixation. (**a**) Schematic depicts the experimental setup. (**b-e**) The same area on a Lec2 cell was probed sequentially before (b,c) and after cell fixation (d,e). FD-based AFM was used to image the height (**b,d**) and to extract the adhesion forces. No significant changes in the binding frequency is observed after fixation. These experiments were repeated independently (N = 5) with similar results.
