# Supplementary material for: Reovirus directly engages integrin to recruit clathrin for entry into host cells
Source: Nat Commun. 2021 Apr 12;12:2149. doi: 10.1038/s41467-021-22380-0 (PMC8041799; doi:10.1038/s41467-021-22380-0)
Supplement: Supplementary file 3 — Description of Additional Supplementary Information [file 41467_2021_22380_MOESM3_ESM.pdf]

Description of Additional Supplementary Files

File Name: Supplementary Movie 1

Description: Time-lapse imaging of clathrin recruitment (BFP, for better visibility shown in green instead of blue) after contact with a T3SA+ virion-coated NP (Alexa647, red) at the plasma membrane. Snapshots of this video are shown in Figure 6b. During real-time imaging, the images were recorded with a frame time of 1.61 s for a total time of ~ 10 min. The movie speed is ~ 16x real-time.

File Name: Supplementary Movie 2

Description: Blocking of clathrin recruitment (BFP, for better visibility shown in green instead of blue) by cRGD after contact with a T3SA+ virion-coated NP (Alexa647, red) at the plasma membrane. Snapshots of this video are shown in Supplementary Figure 6a. During realtime imaging, the images were recorded with a frame time of 1.01 s for a total time of ~ 10 min. The movie speed is ~ 16x real-time.

File Name: Supplementary Movie 3

Description: No clathrin recruitment (BFP, for better visibility shown in green instead of blue) in the presence of Neu5Ac after contact with a T3SA+ virion-coated NP (Alexa647, red) at the plasma membrane. Snapshots of this video are shown in Supplementary Figure 6b. During realtime imaging, the images were recorded with a frame time of 2.01 s for a total time of ~ 10 min. The movie speed is ~ 16x real-time.

File Name: Supplementary Movie 4

Description: No clathrin recruitment (BFP, for better visibility shown in green instead of blue) after contact with a T3SA+ ISVP-coated NP (Alexa647, red) at the plasma membrane. Snapshots of this video are shown in Supplementary Figure 6c. During real-time imaging, the images were recorded with a frame time of 2.01 s for a total time of ~ 10 min. The movie speed is ~ 16x real-time.

File Name: Supplementary Movie 5

Description: No clathrin recruitment (BFP, for better visibility shown in green instead of blue) after contact with a T3SA+ IBM-1-coated NP (Alexa647, red) at the plasma membrane. Snapshots of this

video are shown in Supplementary Figure 7a. During real-time imaging, the images were recorded with a frame time of 2.01 s for a total time of ~ 10 min. The movie speed is ~ 16x real-time.

File Name: Supplementary Movie 6

Description: No clathrin recruitment (BFP, for better visibility shown in green instead of blue) after contact with a T3SA+ IBM-2-coated NP (Alexa647, red) at the plasma membrane. Snapshots of this video are shown in Supplementary Figure 7b. During real-time imaging, the images were recorded with a frame time of 2.01 s for a total time of ~ 10 min. The movie speed is ~ 16x real-time.

File Name: Supplementary Movie 7

Description: No clathrin recruitment (BFP, for better visibility shown in green instead of blue) after contact with an Alexa700 dye-coated NP (red) at the plasma membrane. Snapshots of this video are shown in Supplementary Figure 7c. During real-time imaging, the images were recorded with a frame time of 2.01 s for a total time of ~ 10 min. The movie speed is ~ 16x real-time.
