# Supplementary material for: Reovirus directly engages integrin to recruit clathrin for entry into host cells
Source: Nat Commun. 2021 Apr 12;12:2149. doi: 10.1038/s41467-021-22380-0 (PMC8041799; doi:10.1038/s41467-021-22380-0)
Supplement: Supplementary file 11 — Reporting Summary [file 41467_2021_22380_MOESM11_ESM.pdf]

nature research

Corresponding author(s): Alsteens, David
Dermody, Terence

Last updated by author(s): Feb 17, 2021

Reporting Summary

Nature Research wishes to improve the reproducibility of the work that we publish. This form provides structure for consistency and transparency in reporting. For further information on Nature Research policies, see our Editorial Policies and the Editorial Policy Checklist.

Statistics

For all statistical analyses, confirm that the following items are present in the figure legend, table legend, main text, or Methods section.

n/a	Confirmed	
☐	☒	The exact sample size (n) for each experimental group/condition, given as a discrete number and unit of measurement
☐	☒	A statement on whether measurements were taken from distinct samples or whether the same sample was measured repeatedly
☐	☒	The statistical test(s) used AND whether they are one- or two-sided<br>*Only common tests should be described solely by name; describe more complex techniques in the Methods section.*
☒	☐	A description of all covariates tested
☒	☐	A description of any assumptions or corrections, such as tests of normality and adjustment for multiple comparisons
☐	☒	A full description of the statistical parameters including central tendency (e.g. means) or other basic estimates (e.g. regression coefficient) AND variation (e.g. standard deviation) or associated estimates of uncertainty (e.g. confidence intervals)
☐	☒	For null hypothesis testing, the test statistic (e.g. F, t, r) with confidence intervals, effect sizes, degrees of freedom and P value noted<br>*Give P values as exact values whenever suitable.*
☒	☐	For Bayesian analysis, information on the choice of priors and Markov chain Monte Carlo settings
☒	☐	For hierarchical and complex designs, identification of the appropriate level for tests and full reporting of outcomes
☒	☐	Estimates of effect sizes (e.g. Cohen's d, Pearson's r), indicating how they were calculated

Our web collection on statistics for biologists contains articles on many of the points above.

Software and code

Policy information about availability of computer code

Data collection	Nanoscope Software v9.2 (Bruker), Zen Blue 3.1 (Zeiss), BLItz ProTM software (v1.2, Pall ForteBio)
Data analysis	Nanoscope Analysis v1.9 (Bruker), Origin Software 2019 (OriginLab), GraphPad (Prism 8), ImageJ (v1.52e)

For manuscripts utilizing custom algorithms or software that are central to the research but not yet described in published literature, software must be made available to editors and reviewers. We strongly encourage code deposition in a community repository (e.g. GitHub). See the Nature Research guidelines for submitting code & software for further information.

Data

Policy information about availability of data

All manuscripts must include a data availability statement. This statement should provide the following information, where applicable:
- Accession codes, unique identifiers, or web links for publicly available datasets
- A list of figures that have associated raw data
- A description of any restrictions on data availability

The Source data underlying Figures 2c,g; 3b-e,g,h; 4b,c,e,f,h; 5b,c,e,f,h; Fig. 6c-e and Supplementary Figures 4e,g; 5 are provided as a Source Data file. The full sequence of pT7-S1T3SA+ is available in Genbank accession no. EF494441. Any other listed plasmids encoding reovirus gene segments or mTagBFP2 can be brought commercially at Addgene. All other relevant data are available from the corresponding authors upon reasonable request.

Field-specific reporting

Please select the one below that is the best fit for your research. If you are not sure, read the appropriate sections before making your selection.

- [x] Life sciences
- [ ] Behavioural & social sciences
- [ ] Ecological, evolutionary & environmental sciences

For a reference copy of the document with all sections, see nature.com/documents/nr-reporting-summary-flat.pdf

Life sciences study design

All studies must disclose on these points even when the disclosure is negative.

Sample size	Typically, one hundred data points are needed for single-molecule experiments. Here we are always above this value. Multiple replicates (as stated in the legends of the figures) were tested. Sample size was chosen based on data consistency.
Data exclusions	No data was excluded from the analysis.
Replication	The experiments were sucessfully replicated at least 3-5 times.
Randomization	Not applicable
Blinding	Not applicable

Reporting for specific materials, systems and methods

We require information from authors about some types of materials, experimental systems and methods used in many studies. Here, indicate whether each material, system or method listed is relevant to your study. If you are not sure if a list item applies to your research, read the appropriate section before selecting a response.

Materials & experimental systems

n/a	Involved in the study	
[ ]	[x]	Antibodies
[ ]	[x]	Eukaryotic cell lines
[x]	[ ]	Palaeontology and archaeology
[x]	[ ]	Animals and other organisms
[x]	[ ]	Human research participants
[x]	[ ]	Clinical data
[x]	[ ]	Dual use research of concern

Methods

n/a	Involved in the study	
[x]	[ ]	ChIP-seq
[x]	[ ]	Flow cytometry
[x]	[ ]	MRI-based neuroimaging

Antibodies

Antibodies used	Rat anti-Mouse IgG2a Secondary Antibody, APC, eBioscience (ThermoFisher, 17-4210-82), Alexa Fluor 647-conjugated goat anti-mouse secondary antibody (Abcam, ab150115); α5β1 integrin antibody (P1F6, Abcam, ab177004); antibody against serotype 3 reovirus sigma1 protein (9BG5, Sigma, MAB994)
Validation	The antibodies were validated by the manufacturer as stated on the manufacturers website. The primary antibody 9BG5 was validated in a previous study (Dietrich et al., J Virol. 2017 ; 91(4): e01621-16.)

Eukaryotic cell lines

Policy information about cell lines

Cell line source(s)	Lec2 (ATCC® CRL-1736); L929 (ATCC® CCL-1); CHO-K1 (ATCC® CCL-61) (used for producing over-expressing CHO-JAM-A cells)
Authentication	The cells obtained from ATCC were authenticated prior to purchase using STR profiling. The replicates of each experiment were conducted with cells from different passages, and no significant difference was observed among these replicates.
Mycoplasma contamination	We did not see any indicators of mycoplasma contamination.
Commonly misidentified lines (See ICLAC register)	The cell lines used are not listed as commonly misidentified in the ICLAC register.
